# Investigating the Anti-Inflammatory Activity of Various Brown Algae Species

**DOI:** 10.3390/md22100457

**Published:** 2024-10-05

**Authors:** Selin Ersoydan, Thomas Rustemeyer

**Affiliations:** 1 Faculty of Medicine, University of Groningen, University Medical Center Groningen, 9713 GZ Groningen, The Netherlands; 2 Amsterdam University Medical Center, 1007 MB Amsterdam, The Netherlands

**Keywords:** brown algae, anti-inflammatory, fucoidans, polyphenols, dermatology

## Abstract

This literature review investigated the anti-inflammatory properties of brown algae, emphasizing their potential for dermatological applications. Due to the limitations and side effects associated with corticosteroids and immunomodulators, interest has been growing in harnessing therapeutic qualities from natural products as alternatives to traditional treatments for skin inflammation. This review explored the bioactive compounds in brown algae, specifically looking into two bioactive compounds, namely, fucoidans and phlorotannins, which are widely known to exhibit anti-inflammatory properties. This review synthesized the findings from various studies, highlighting how these compounds can mitigate inflammation by mechanisms such as reducing oxidative stress, inhibiting protein denaturation, modulating immune responses, and targeting inflammatory pathways, particularly in conditions like atopic dermatitis. The findings revealed species-specific variations influenced by the molecular weight and sulphate content. Challenges related to skin permeability were addressed, highlighting the potential of nanoformulations and penetration enhancers to improve delivery. While the in vivo results using animal models provided positive results, further clinical trials are necessary to confirm these outcomes in humans. This review concludes that brown algae hold substantial promise for developing new dermatological treatments and encourages further research to optimize extraction methods, understand the molecular mechanisms, and address practical challenges such as sustainability and regulatory compliance. This review contributes to the growing body of evidence supporting the integration of marine-derived compounds into therapeutic applications for inflammatory skin diseases.

## 1. Introduction

Inflammation is a common pathophysiological process underlying many skin diseases such as acne, psoriasis and dermatitis. Immune responses can start inflammatory cascades that can lead to tissue damage and exacerbate skin conditions. Current treatment strategies for inflammatory dermatological conditions, such as atopic dermatitis and psoriasis, consist of topical agents like corticosteroids and calcineurin inhibitors, systemic medications, phototherapy and biologicals [1,2]. However, long term use of these medications comes with several downsides such as skin atrophy, skin irritation, immunosuppression, increased susceptibility to infections and systemic side effects such as nausea and vomiting [2,3]. In addition to these challenges, the high costs of certain treatments and variable patient response to treatment emphasise the need for new effective alternatives with fewer side effects [1,4].

Given the limitations of conventional treatments mentioned above, there is growing interest within the field of dermatology to research alternative therapies. Natural products are an attractive source that can be used in treatments given their high safety profiles, sustainability and lower costs [5]. Marine life stands out as a great candidate to explore as they are rich in biologically active substances with qualities that can be applied to skin pharmaceuticals [6]. These biochemical constituents within marine compounds are known to have outstanding potential as anti-inflammatory and antioxidant agents [7,8]. Their wide range of pharmacological effects make them promising candidates for the development of effective treatments for inflammatory skin diseases [8].

Specifically, brown algae have been of interest for many researchers in the field of dermatology. Their rich bioactive content including fucoidans, polyphenols, and other secondary metabolites known for their ability to alleviate inflammation have been explored [8,9]. These properties have sparked significant hope for their use in treating inflammatory skin diseases such as atopic dermatitis, acne and psoriasis. The long history of the use of algae-derived compounds in traditional medicine to treat skin diseases, alongside their safety profile, makes them attractive candidates for cosmeceutical product development [8]. Research to date has focused on understanding the chemicals produced by various species of algae and explored if and how these can influence inflammatory pathways and skin conditions. These investigations seek to garner knowledge to be used in developing new and efficient treatments that can reduce inflammation, decrease proinflammatory markers, and modulate inflammatory cytokines.

This literature review aimed to investigate the anti-inflammatory properties of various species of brown algae. Determining whether brown algae have anti-inflammatory properties, comprehending the mechanisms underlying this possible activity, and identifying possible compounds that may be responsible for these effects are the main research questions driving this research. To examine these compounds’ ability to permeate skin—a critical component in the development of topical applications—their sizes are also being evaluated. Topical drug delivery has been chosen for the purpose of this study as it comes with several advantages, including its ability to target the desired site, avoid first-pass metabolism, boost bioavailability and prevent systemic side effects [10].

By addressing these objectives, this literature review seeks to advance our understanding of the therapeutic potential of brown algae in dermatology and open the door to the creation of novel, potent skincare solutions harnessing the benefits of marine resources.

## 2. Results

In this literature search, a total of 26 different brown algae species (Table 1) were investigated, with a focus on identifying and analysing their bioactive compounds, such as fucoidans, phlorotannins, and others, which contribute to their therapeutic potential.

### 2.1. Chemical Composition

Brown algae, while varying in their specific compounds across species, share several common chemical characteristics. The pigment fucoxanthin is responsible for the algae’s characteristic brown colour. Cell wall components including fucoidan, a sulphated polysaccharide, alginate, a structural polysaccharide, and cellulose are found in all brown algae. Laminarin, a β-glucan storage polysaccharide, and mannitol, a sugar alcohol, are part of their storage components. Lastly, brown algae are known to produce secondary metabolites, which include phlorotannins, a polyphenolic compound, and phytosterols, such as fucosterol.

Brown algae’s chemical profiles provide insight into their rich array of bioactive components. To determine the chemical composition of these algae, several chemical extraction techniques were used.

Fucoidans and polyphenols, specifically phlorotannins, were among the most common constituents of brown algae. The anti-inflammatory and antioxidant properties of these compounds make brown algae a valuable resource for dermatological treatments. Fucoidans, which are sulphated polysaccharides, are primarily composed of a carbohydrate called l-fucose. L-fucose has an integral role in the therapeutic effects of brown algae as it is known to have potent anti-inflammatory properties. The sulphated nature of fucoidans, found in species such as *Sargassum siliquosum*, is critical for their biological activity as it enhances their ability to ameliorate inflammation [11].

High-resolution liquid chromatography-mass spectrometry quadrupole time-of-flight (HRLCMS QTOF) analysis was used to further identify various other compounds within the brown algae *Padina boergesenii* and *Carpomitra costata* such as phlorotannins, fatty acid derivatives, peptides, terpenoids, and amino acids—all commonly sharing antioxidant and anti-inflammatory properties [12,13].

In addition, 1H NMR further found compounds such as sugars, pigments and lipophilic chemicals in the algae *Saccorhiza polyschides* [14]. Among these pigments are fucoxanthin, phycoerythrin, phycocyanin, carotenoids, and chlorophylls, which not only contribute to the algae’s colour but also offer various health benefits [14].

Fourier-transform infrared analysis also revealed the presence of acids, alcohols, esters, amines and halo compounds, broadening the spectrum of potentially therapeutic substances found in brown algae [14]. Phytol was a specifically interesting finding, as this alcohol has been proven to have anti-inflammatory properties in other studies.

In a study using gas chromatography-mass spectroscopy, hydrocarbon derivatives, fatty acids, fatty esters, and long-chain fatty alcohols were identified [12].

It is evident that the chemical composition of brown algae is rich and thus shows great potential in providing potentially useful applications in skincare and dermatology that extend beyond their anti-inflammatory properties.

#### 2.1.1. Fucoidans

Fucoidans are among the most abundant compounds across various species of brown algae. They are primarily composed of fucans, fucoglucans and fucoxylands. According to research, properties of fucoidans can vary from species to species, thus leading to different degrees of anti-inflammatory qualities. The anti-inflammatory properties of fucoidans seem to be related to their ability to prevent protein denaturation and preserve the integrity of human red blood cell (HRBC) membranes, which are essential for maintaining cellular stability and function [15]. Inflammation suppression happens largely due to the high sulphate content of fucoidans. This happens because sulfation strengthens the biological activity of these polysaccharides, thus enabling them to inhibit inflammatory processes more effectively. In addition to sulphate, the polyphenol content and antiradical activity of fucoidans also greatly contribute to their anti-inflammatory properties [11,15]. Fucose, a key carbohydrate within fucoidans, has shown a positive correlation with inflammation reduction, further supporting the therapeutic potential of these compounds. These compounds show strengthened properties when present together. The optimal combination for the highest anti-inflammatory effects is high amounts of carbohydrates, high sulphate levels and a low molecular weight, resulting in a greater ability of protein denaturation and hence reduced inflammation. One mechanism attributed to inhibiting protein denaturation was through modifying autoantigen synthesis. This, in turn, prevents autoimmune responses that can exacerbate inflammatory conditions [16].

In a study, fucoidans derived from the species *Dictyota menstrualis* and *Fucus vesiculosus* have shown therapeutic potential in ameliorating symptoms of atopic dermatitis, which include inflammation, dryness and itching [16]. These fucoidans seem to exert their effect by modulating the immune response through activating regulatory T cells (Tregs). This plays a critical role in controlling inflammation and maintaining immune homeostasis [16].

#### 2.1.2. Polyphenols

The main phenolic compounds found in brown algae are phlorotannins, catechins, epicatechins, bromophenols, flavonoids, and phenolic terpenoids. Phlorotannins, the most important type of polyphenol found in brown algae, are another group of compounds that contribute significantly to the anti-inflammatory properties of brown algae. Reactive oxygen species contribute to initiating and exacerbating inflammation as well as promote cellular damage. Phlorotannins exert their effects by inhibiting these reactive oxygen species [13,17,18]. In this way, phlorotannins prevent oxidative stress and stop one of the mechanisms of inflammation and damage. In addition to direct anti-inflammatory effects, phlorotannins have been found to further contribute to skin health by inhibiting enzymes such as collagenase and elastase. Excessive activity of these enzymes leads to reduced skin elasticity, degradation of the skin structure and function, as well as impaired wound healing. Elastase is also often released during inflammatory skin conditions. This results in exacerbation of skin symptoms and worsens conditions like acne, atopic dermatitis, and psoriasis. By inhibiting these enzymes, phlorotannins not only reduce inflammation but also help to maintain a healthy skin structure, making them valuable components in skincare formulations [13,19].

### 2.2. Proinflammatory Cytokines

Several studies have explored the effects of brown algae on pro-inflammatory cytokines. These are signalling molecules that mediate and regulate immune responses and promote the development of inflammation. These cytokines are involved in the pathogenesis of various inflammatory skin conditions, including atopic dermatitis, psoriasis, and acne. Many of these studies revealed their capabilities of decreasing pro-inflammatory cytokines.

*Saccorhiza polyschides* is a good example; it has exhibited a significant reduction in TNF-α and IL-6 levels [14]. This reduction in proinflammatory cytokine levels suggests that brown algae could be effective in alleviating the symptoms of inflammatory skin diseases by dampening the immune response that leads to these conditions. However, not all species of brown algae exhibit the same degree of inhibition. *Dictyota menstrualis* showed little impact on the release of TNF-α, IL-1β, and IL-6, indicating that the anti-inflammatory effects of brown algae can show variations depending on the species that is being used [20]. In vivo studies have provided further evidence of the anti-inflammatory effects of fucoidans. These studies have shown that fucoidan treatment can lead to a decrease in pro-inflammatory cytokines such as TNF-α and IL-12, both of which are key mediators of inflammation in the body. Additionally, in vitro studies using brown algae extracts have demonstrated a significant reduction in the release of pro-inflammatory mediators like TNF-α, as well as PGE2, IL-6, and IL-1β. These findings highlight the potential of brown algae to modulate immune responses and reduce inflammation in a variety of settings [16,21].

*Padina crinita* is another brown algae species that was investigated for its effectiveness in reducing pro-inflammatory cytokines. These extracts resulted in substantially reduced PGE2 levels when compared to positive control groups. The same study also reported reduced expression of COX-2 and IL-1β [22]. Similarly, studies using both freeze-dried *Halopteris scoparia* extract and alkaline hydrolysis-derived *Halopteris scoparia* extract found they led to inhibition of IL-6 and TNF-α [23].

### 2.3. Anti-Inflammatory Cytokines

The ability of brown algae to promote the production of anti-inflammatory cytokines has also been studied. Unlike pro-inflammatory cytokines, the presence of anti-inflammatory cytokines is favourable as these are involved in the inhibition of inflammatory pathways, suppress the activities of immune cells involved in inflammatory responses, and stimulate tissue repair.

One study found that fucoidan treatment induced the production of key anti-inflammatory cytokines, including IL-4, IL-10, and TGF-β [16]. In a different study, however, *Saccorhiza polyschides* fractions were unable to stimulate the production of the anti-inflammatory cytokine IL-10. Once again, the differing properties of compounds extracted from different species of brown algae seem to have varying effects on the release of anti-inflammatory cytokines, highlighting the complexity of brown algae [14].

### 2.4. In Vivo Anti-Inflammatory Effects

The anti-inflammatory properties of brown algae have been supported by various in vivo studies. Carrageenan-induced paw oedema is one of the models that has been used to research and study acute inflammation. The carrageenan-induced paw oedema model is widely used and well-characterized for studying acute inflammation as this model mimics acute inflammatory conditions, making it physiologically relevant for studying inflammation and testing anti-inflammatory agents. In multiple studies, a significant reduction in carrageenan-induced paw oedema in rats has been observed following treatment with brown algae extracts [17,24,25].

A dose-dependent correlation seems to underly the anti-inflammatory effects of brown algae given that higher doses of algae extracts were associated with greater reductions in paw oedema. This suggests that optimal therapeutic effects can only be achieved once the correct dose has been determined [24,25]. Additionally, similar dose-dependent anti-inflammatory effects were observed in the xylene-induced ear oedema model [17].

These studies demonstrate a high degree of specificity in their experimental designs, focusing on particular brown algal species and their unique chemical compositions. One of the studies further refined the specificity by isolating phlorotannin-rich fractions from three Mediterranean brown seaweeds, demonstrating how particular chemical components contribute to biological activities [17].

### 2.5. Antioxidant Effects

Free radicals are commonly thought to be a trigger for inflammation by causing oxidative stress, triggering the release of pro-inflammatory cytokines, activating inflammatory pathways and leading to tissue damage. Therefore, compounds that target free radicals are considered optimal agents for mitigating inflammation. Brown algae are known to have antioxidant effects, which target this mechanism of inflammation [16].

Fucoidans are particularly interesting in this regard. Different species of algae containing fucoidans have shown varying levels of antioxidant activity and thus varying levels of anti-inflammatory effects. One study found that higher levels of sulphate content in fucoidans were associated with greater reductions in superoxide radicals, a type of free radical that contributes to oxidative stress and inflammation [11].

Alongside fucoidans, phlorotannins also exhibit strong antioxidant activity, as identified by radical scavenging assays [17].

### 2.6. Mechanisms Contributing to Anti-Inflammatory Effects

#### 2.6.1. Protein Denaturation

Protein denaturation is a process in which a protein loses its structure and consequently also its function. This denatured protein can then lead to inflammation by triggering an immune response. They can also produce reactive oxygen species, which leads to exacerbated tissue damage. Another mechanism of fucoidans and phlorotannins that helps reduce inflammation is through its ability to inhibit protein denaturation [15]. This inhibition is particularly important as protein denaturation plays a role in the progression of several inflammatory skin diseases. Studies have shown that the inhibition of protein denaturation by phlorotannins is concentration-dependent, suggesting that higher concentrations may be more effective at reducing inflammation [18].

#### 2.6.2. Inhibition of Nitric Oxide

Nitric oxide is a key inflammatory mediator involved in various inflammatory processes. To investigate the anti-inflammatory properties of brown algae, the levels of nitric oxide in RAW 264.7 cells were tested and the inhibitory effects were determined. The results of these studies produced varied outcomes, with some studies showing no stimulation of nitric oxide production [13,14,19], while a different study observed no effect on the reduction of nitric oxide levels induced by treatment with lipopolysaccharides [13]. Interestingly, one study showed an increase in nitric oxide levels induced by treatment with lipopolysaccharides. These results suggest that the anti-inflammatory properties of algae might not solely rely on the suppression of nitric oxide and other mechanisms may underlie their anti-inflammatory effects [14].

The variability in these results highlights the complexity of natural biomass. It is possible that a number of variables might affect the bioactivities of natural biomass and thus other factors might also have an influence on how much nitric oxide production is inhibited.

#### 2.6.3. Anti-Inflammatory Pathways

The NF-κB and MAPK signalling pathways are important components of the inflammatory response. Both of these pathways lead to the synthesis of pro-inflammatory mediators, thus making them key targets for anti-inflammatory therapies. One study found that algae-derived compounds can inhibit the activation of the NF-κB and MAPK pathways, thereby reducing the expression of pro-inflammatory mediators [21]. Furthermore, it has been found that the production of Tregs and upregulation of the anti-inflammatory cytokines IL-10 and TGF-β can be triggered by fucoidans. This specific mechanism has been shown to relieve the symptoms of atopic dermatitis [16].

Together, these findings suggest possible therapeutic routes for the management of inflammation and thus the development of targeted drugs.

### 2.7. Toxicity

The safety profile of using brown algae is a critical consideration in creating novel therapies. The toxicity of brown algae was investigated using RAW 264.7 cells, and the results were generally positive. Brown algae have shown no cytotoxicity, indicating their safety for use in therapeutic applications [26].

### 2.8. Dermal Effects

A subset of research has focused exclusively on dermatological problems. Fucoidan treatment of mice with DNFB-induced atopic dermatitis produced significant results as seen by decreased dermatitis scores and reduced symptoms such as swelling. These findings suggest that fucoidan could be a promising therapeutic intervention for atopic dermatitis. Moreover, fucoidan therapy significantly reduces the increased epidermal thickness and inflammatory cell infiltration in the ears of these animals. This further supports its potential in managing skin conditions characterized by inflammation and hyperproliferation [16].

### 2.9. Permeability through Skin

#### Molecular Size

To ensure the efficacy of a topical dermatological drug, there must be good skin permeability. Molecular weight, lipophilicity, and molecular charge are all factors that influence the ability of a compound to pass through the skin. According to the “500 Dalton rule”, molecules larger than 500 Daltons cannot pass through the surface layer of the skin, limiting their effectiveness as topical agents [27].

For the scope of this literature search, the molecular weight of fucoidans and polyphenols, specifically phlorotannins, was investigated. The molecular size of fucoidans ranges from 13 to 950 kDa, which suggests that many of these molecules are too large to permeate the skin effectively [28,29]. Phlorotannins, with a molecular weight range between 10 to 100 kDa, may have better skin permeability, although this depends on their specific structure and lipophilicity [30].

One way to tackle the challenge of reduced permeability can be by making use of lipid-soluble formulations. These formulations enhance the ability of large molecules to penetrate the skin by increasing their lipophilicity, thereby improving the ability of the drug to reach the deeper layers of the skin, where it can exert its therapeutic effects [31,32].

## 3. Discussion

Explorations of the chemical composition of brown algae have shown a diverse range of bioactive components. Fucoidans and polyphenols are the most prominent compounds in brown algae and offer great potential in dermatological applications [11,13,15,16,17,18,19].

Fucoidans in particular display species-specific anti-inflammatory effects.

Species with a low molecular mass, enabling easier penetration through skin, or with high sulphate levels and high carbohydrate levels, demonstrated a stronger ability to lower oxidative stress, inhibit protein denaturation and stabilize human red blood corpuscle (HRBC) membranes, all potential targets for treating inflammatory skin conditions. Sulfation enhances the anti-inflammatory effects of compounds like fucoidans by increasing their negative charge, which improves their ability to bind to and neutralize positively charged inflammatory mediators, such as cytokines and enzymes. This prevents the activation of the inflammatory response. Sulphated compounds also block leukocyte adhesion to endothelial cells, reducing immune cell migration to inflamed tissues. Additionally, sulfation inhibits pro-inflammatory signalling pathways, such as NF-κB, further suppressing the production of inflammatory proteins. These combined actions make sulphated compounds highly effective in reducing inflammation. Future use of fucoidans in treatment of dermatological disorders could focus on tailoring the molecular mass, sulphur, and carbohydrate contents. Fucoidans derived from *Dictyota menstrualis* and *Fucus vesiculosus* exhibited therapeutic effects in ameliorating symptoms of atopic dermatitis, suggesting promising potential as an alternative treatment to the currently existing topical steroid creams [11,22].

The ability of fucoidans to penetrate the skin to be able to produce therapeutic effects is an important discussion point. The high molecular weight, negative charge, and hydrophilicity of fucoidans can lead to a poor ability to penetrate the skin. One way to overcome these challenges is through the use of ointments containing penetration enhancers, such as Transcutol, which in combination with fucoidans has been effective in boosting skin penetrability [29]. Using nanoformulations is another innovative way that can further enhance the delivery of compounds like fucoidans. The particle sizes in these formulations range from 10 to 1000 nm, making them small enough to penetrate through the skin barrier. This ensures improved drug absorption and targeted delivery by allowing the active compounds to reach the desired tissue while minimizing systemic exposure and side effects [33]. In addition to the aforementioned techniques, microemulsions and liposomes can also be used for topical delivery systems as they enhance the solubility of both hydrophilic and lipophilic compounds. However, care must be taken with these formulations. Components such as fatty acids and surfactants that are found in these microemulsions and liposomes can disrupt the skin barrier, potentially leading to exacerbation of inflammatory conditions. It is therefore crucial to balance enhancing the skin permeability while also maintaining barrier integrity [6].

It is also important to take into account the complex interplay between choosing a high enough dose to produce the desired biological effects while also avoiding toxic doses. While some studies in this literature search have found no cytotoxic effects of brown algae [26], specific tests for fucoidans still remain open to research. The correct therapeutic dosage should be researched in the future in accordance with each specific inflammatory disease.

Phlorotannins identified in brown seaweeds also demonstrated anti-inflammatory effects [13,17,18,19]. The extraction methods used have shown to influence the bioactive properties of phlorotannins. Different methods result in different compositions of the phlorotannin extracts. This in turn highlights an important point of consideration while designing products for the treatment of inflammatory skin diseases. Ethyl acetate has shown to be a good solvent for extraction, where phenols exhibited the highest free radical scavenging activity [14,18]. Advanced extraction techniques such as ultrasonic-assisted extraction (UAE) and microwave-assisted extraction (MAE) offer significant improvements in the efficiency and yield of compounds from brown algae. UAE makes use of high frequency sound waves to extract polysaccharides and phenolic compounds while significantly reducing the extraction time compared to conventional methods [34,35]. MAE, on the other hand, applies microwave energy to extract the algal material, leading to rapid extraction of bioactive compounds and improved yields of polysaccharides and antioxidants [34,35]. Another method is by using acidic and alkaline extractions. Acid extraction has been effective in yielding high concentrations of fucoidan, while alkaline conditions improve the solubility of polysaccharides [36]. The potency of algal extracts can increase with the employment of these advanced extraction methods. In this way, the efficacy of these topical treatments could be significantly improved.

Once again, it is important to take into consideration the permeability of phlorotannins through skin. While molecular weight is a contributor in predicting permeability, the physicochemical and molecular properties, polarity, the proteins and carbohydrates as well as carrier substances all have an influence. Applications using ointment seem to be an advantageous choice [32]. Similarly to fucoidans, more investigation is necessary to identify targeted therapeutic dosages.

Many of the algae in the studies demonstrated the ability to inhibit pro-inflammatory cytokines such as PGE2, TNF-α, IL-6, and IL-1β [14,20,21,22,23] as well as stimulate anti-inflammatory cytokines such as IL-4, IL-10 and TGF-β [14,17]. These results suggest that brown algae can play an important role in interrupting inflammatory cascades and thus hold great promise for the development of treatments against skin diseases such as atopic dermatitis, psoriasis, eczema and acne, which are characterized by disrupted immune responses and increased inflammation.

In vivo studies have added a new dimension to these promising findings [17,24,25]. Carrageenan-induced paw oedema is a widely used experimental model that mimics acute inflammatory responses similar to those seen in various skin conditions, such as dermatitis, psoriasis, and eczema. The reduction in paw oedema shows that the compounds in brown algae, such as fucoidans and phlorotannins, are capable of suppressing inflammation and therefore can have applications in treating dermatological skin diseases. Fucoidans already show positive results in ameliorating atopic dermatitis in mice [16]. Understanding the detailed mechanisms underlying the therapeutic effects of brown algae can enable tailoring treatment specific to a particular skin disease while also allowing the possibility of comparisons with existing therapies. Clinical trials should be used in future research to evaluate the efficacy and safety of new therapies, and these should be compared to the currently existing ones. Possible side effects and long-term repercussions of therapy employing chemicals inside brown algae should be established.

The anti-inflammatory signalling pathways have not been thoroughly explored. One article has demonstrated the suppression of the NF-κB and MAPK signalling pathways’ activity, which is an important pathway for several inflammatory skin diseases [21]. Other studies not included in this literature review have also confirmed this finding and delved further into explaining the underlying mechanisms. It has been found that the inhibition of the NF-κB pathway occurs through mechanisms such as blocking the phosphorylation of IKK-α and IKβ-α, which are protein kinases involved in this signalling pathway and are central to controlling the immune response. Furthermore, fucoidans downregulate the expression of NF-κB target genes like iNOS and COX-2, supporting the anti-inflammatory effects noted in earlier studies [37,38,39]. Moreover, fucoidans have been shown to inhibit the phosphorylation of certain protein kinases involved in pathways of inflammation and reduce the activation of other kinases in inflammatory cells [15]. Anti-inflammatory cascades of phlorotannins have also been studied and similar inhibition pathways have been found. Phlorotannins not only inhibit the NF-κB pathway, reducing the expression of inflammatory genes, but also disrupt the cascades within the MAPK pathways. In addition, phlorotannins inhibit key enzymes like secretory phospholipases A2, lipoxygenases, and cyclooxygenase-2, all of which are involved in the production of pro-inflammatory mediators [40,41,42]. Investigating signalling pathways, relationships with inflammatory mediators, and the effect on immune cells can help in the development of targeted therapies. Identifying specific bioactive components and separating them from the different algae extracts is also crucial for understanding the functions of the varying compounds, explaining their effects and comprehending their role in tailored treatments. The compound–activity relationships should therefore be further explored. However, as seen with fucoidans, the complexity of these natural extracts emphasizes the challenges in identifying their bioactive components.

While the scope of this literature review was to explore the anti-inflammatory effects of brown algae, it is also noteworthy to mention additional properties that can help in alleviating inflammatory skin diseases and improve skin health. Moisturizers are a mainstay of treatment for inflammatory skin disorders such as atopic dermatitis. Daily use of moisturizers containing emollients, humectants, and occlusive ingredients improves barrier function and reduces the need for topical corticosteroids. The bioactive compounds within brown algae seem to also help with skin hydration and wound healing. Alginate is a polysaccharide found in algae and it has excellent water-retaining properties, making it highly effective for skin hydration and moisturization. This is particularly beneficial for patients with atopic dermatitis, where maintaining skin moisture helps preserve the skin’s barrier function. Alongside hydration, alginates also have wound healing properties and promote tissue regeneration. Given that chronic wounds are commonly associated with conditions such as atopic dermatitis, alginates seem to be a good therapeutic option in managing such skin problems [43]. Extracts from the algae species *Laminaria japonica* have also shown the ability to moisturize and thus help strengthen the skin barrier [44,45]. Another complication that arises in atopic dermatitis is an increased risk of bacterial skin infections, in particular those caused by *Staphylococcus aureus*. This is due to trauma to the skin due to scratching together with a compromised skin barrier. Additionally, there is a significant decrease in skin microbial diversity, which creates room for pathogenic bacteria like *Staphylococcus aureus* to become more dominant and leads to an increased disease severity. This highlights the need for treatments that can restore microbial diversity and control pathogenic bacteria [46]. Various algae species, namely those belonging to the groups *Sargassum* and *Padina*, have demonstrated the ability to kill harmful fungi and bacteria, helping to maintain a balanced skin flora [47]. For instance, the algae species *Sargassum macrocarpum* has been shown to be effective against acne by targeting the bacteria *Propionibacterium acnes*, which is responsible for the disease pathogenesis. Other Sargassum species have been effective against both Gram-positive and Gram-negative bacteria [48,49]. Additionally, fucoidans from brown algae such as *Fucus vesiculosus* have shown significant ability in preventing bacterial growth, especially against strains like *E. coli*, *S. epidermidis*, *S. aureus*, and *B. licheniformis*, further supporting their potential as antimicrobial agents [50]. These antimicrobial properties extend to other brown algae species as well. The methanolic extracts of *Stypopodium schimperi*, *Halopteris filicina*, *Dictyota dichotoma*, *Gracilaria bursa-pastoris*, and *Ulva intestinalis* have shown effectiveness against a range of pathogenic bacteria [51].

The therapeutic potential of brown algae-derived compounds like fucoidans and phlorotannins is significant. However, to be able to harness the benefits of these algae, it is important to consider the practical and logistical limitations associated with the production of marine-derived products.

One point of attention is the presence of contaminants that occur naturally in marine environments. These can include heavy metals such as cadmium, lead, arsenic, and mercury, and therefore pose a risk to human health. To ensure a high safety profile and minimize the toxicity of these products, it is of utmost importance to remove such harmful substances.

Another challenge to tackle is maximizing the volume of algae produced, as these are generally present in only small quantities in nature.

Alongside these points, it is noteworthy to consider the effects of variable environmental conditions on the compounds within algae. Different conditions can lead to differing amounts and qualities of the metabolites, hence altering their therapeutic properties. To establish consistency of the bioactive products, it is crucial that the algal extracts are standardized by ensuring stable environments and optimal extraction methods. Sourcing these compounds is also challenging, as some of the target algae species are located in difficult to access locations, like deep in ocean floors [52].

Considering methods of eco-friendly and sustainable harvesting is also pivotal when harvesting algae. Over-exploitation and damage to the ecosystem due to large-scale cultivation should be prevented.

Ensuring that the bioactive compounds are stable and have a long shelf life is also important when creating products. Bioactive compounds from marine algae are prone to oxidation and degradation over time, which can affect the efficacy of the final product [43]. When creating a product with multiple ingredients, interactions must also be carefully studied in order to ensure that safety and efficacy are not compromised.

Given the novelty of such natural treatment modalities, people might have a sceptical approach. Educating consumers about the benefits and safety of these products is essential to encourage the use of such treatments [43].

Regulatory compliance presents another layer of complexity. Navigating the regulatory frameworks to ensure that algae products meet safety and efficacy standards will allow for greater acceptance among consumers.

Finally, establishing the cost and scalability of producing high-quality marine algae extracts must be explored. Addressing the cost of production and ensuring the scalability of these extracts are crucial for making them a realistic treatment option in the long term [43].

## 4. Future Directions

Investigating the anti-inflammatory effects of brown algae has revealed a diverse range of bioactive components with significant therapeutic potential, particularly in the field of dermatology. Together with these results, several avenues for future exploration have opened. Addressing these topics can allow for a holistic approach to be able to fully harness the potential of these marine-derived compounds.

### 4.1. Optimization of Extraction Techniques

One of the key areas for future research is the optimization of extraction methods for fucoidans, phlorotannins, and other bioactive compounds found in brown algae. Advanced techniques such as ultrasonic-assisted extraction (UAE) and microwave-assisted extraction (MAE) have shown to be good options for improving yield and efficiency. The most suitable extraction methods must be identified such that consistent and scalable production is achieved. By creating standardized protocols, the variability in extraction outcomes, particularly with different algae species and environmental conditions, can be avoided. This would ensure consistent and reliable products that can easily be reproduced across different batches.

### 4.2. Enhancing Skin Penetrability

The therapeutic efficacy of fucoidans and phlorotannins is closely linked to their ability to penetrate the skin. Given the challenges posed by the high molecular weight and hydrophilicity of these compounds, especially of fucoidans, future research should focus on developing delivery systems allowing these compounds to reach the deeper layers of the skin. Nanoformulations have been explored in this review and they could be further explored to curate enhanced drug absorption and targeted delivery methods. Additionally, the use of penetration enhancers such as Transcutol should be further explored to balance the need for improved skin permeation with the preservation of skin barrier integrity. The safety of these delivery methods must be thoroughly assessed and their efficacy must be evaluated.

### 4.3. Targeted Therapeutic Dosages

Like any other therapeutic drug, optimal dosages of brown algae products to achieve maximal disease mitigation must be established. Furthermore, toxic doses must also be identified. Research should focus on understanding the dose–response relationship for different compounds and be adjusted for different inflammatory skin diseases. This will involve not only preclinical studies but also carefully designed clinical trials to validate the findings in human populations.

### 4.4. Expanding the Scope of Anti-Inflammatory Research

While the current research has provided valuable insights into the anti-inflammatory mechanisms of fucoidans and phlorotannins, the pathways involved are not yet fully understood. While key signalling pathways such as NF-κB and MAPK have been explored, the identification of disease-specific mechanisms can allow for a more targeted approach when creating treatments.

### 4.5. Addressing Practical and Logistical Challenges

The practical challenges associated with the production and use of marine-derived ingredients must be addressed to bring these compounds from the laboratory to the market. Ensuring that hazardous contaminants such as heavy metals do not end up in the final product is pivotal. Additionally, the scalability of production, environmental variability, and methods of creating stable growth conditions as well as the need for sustainable harvesting practices requires further investigation. Research should focus on developing sustainable methods for cultivating and harvesting brown algae, as well as on improving the stability and shelf life of bioactive compounds in the final product formulations.

### 4.6. Consumer Education and Regulatory Compliance

For the successful introduction of marine algae-derived products into the market, these should pass extensive regulatory checks. Meeting complex regulatory standards will require extensive testing to ensure that these products meet all necessary safety and efficacy standards. This will involve close collaboration with regulatory bodies and continuous monitoring of emerging guidelines.

Furthermore, consumers should be educated about these products, preferably by healthcare specialists. Future research should explore the most effective ways to educate consumers about the benefits and safety of these products, addressing any scepticism that may exist.

### 4.7. Clinical Studies

To ensure the safety and efficacy of brown algae products for humans, extensive clinical trials must be carried out. These studies should investigate whether therapeutic effects against inflammatory skin diseases are observed and compare the effectiveness with existing therapies like topical corticosteroids.

### 4.8. Market Viability

Research should focus on the economic aspects of production. While brown algae products can be promising dermatological treatments, it is crucial that the costs allow these to be easily accessible for patients and be commercially viable.

### 4.9. Different Algae Species and Their Chemical Composition

This research has repeatedly mentioned that anti-inflammatory effects can be species-specific. While there are several chemical components that overlap across all brown algae types, there seem to be significant differences in the composition of these compounds between different species. Future research could focus on identifying the characteristic chemical compositions of particular brown algae species and how these contribute to their differing therapeutic effects.

## 5. Materials and Methods

The articles reviewed were selected from PubMed databases in the period 2013–2023. The MeSH terms “anti-inflammatory”, “brown algae”, and “seaweed” were used. The choice of articles was made based on the relevance of the topic, research objectives, results and year of publication. This review includes in vitro and in vivo studies. A total of 17 articles were selected.

### 5.1. Inclusion and Exclusion Criteria

The inclusion criteria were articles written in English in the period 2013–2023 that investigated the anti-inflammatory properties of brown algae species, reporting specific anti-inflammatory outcomes, such as reduced inflammation, inhibition of pro-inflammatory markers, or modulation of cytokine levels and in vitro, in vivo, or clinical trial designs.

**Figure 1:**
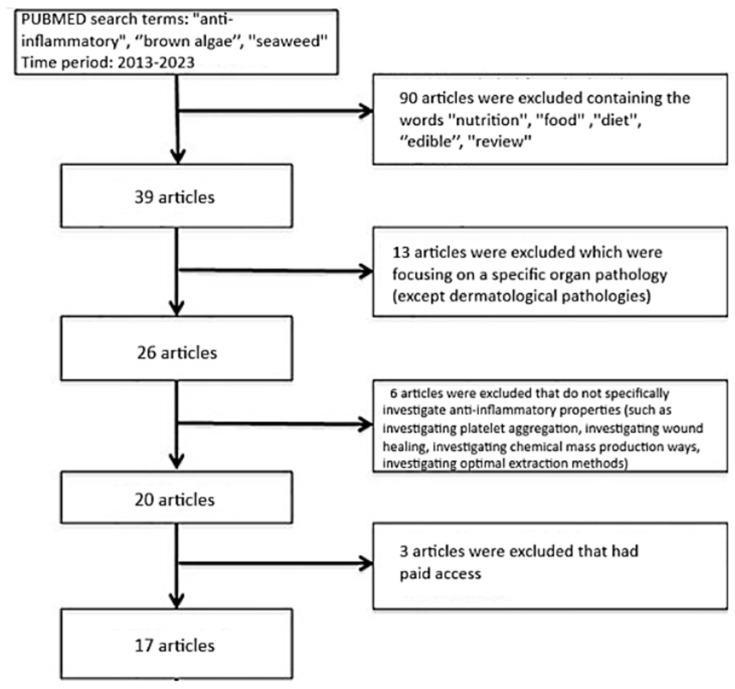
Inclusion and exclusion criteria.

Exclusion criteria (Figure 1) were studies on edible algae and a focus on algae as part of food, diet and nutrition.

### 5.2. Critical Appraisal

All studies were evaluated based on their research objectives, methodology and relevant conclusions. There were no identified risks of bias or conflicts of interest.

### 5.3. Data Extraction

Per study, the algae species and compounds investigated, study design, anti-inflammatory assays, and outcome measures were extracted and collected in Table 2.

## 6. Strengths and Limitations of the Literature Study

### 6.1. Strengths

The literature exhibits commendable utilization of diverse analytical approaches, employing a range of assays for a comprehensive evaluation of brown marine algae’s anti-inflammatory potential. Furthermore, the inclusion of detailed experimental procedures is noteworthy, providing explicit methods for extraction, purification, and assays, enhancing the study’s transparency and reproducibility. Additionally, this research displays a strength in its exploration of a large number of algae species, ensuring a comprehensive analysis and a broad understanding of potential anti-inflammatory properties across diverse species.

### 6.2. Limitations

The reliance on a single study source, with samples collected from a specific location during a limited or single time frame, raises concerns about the generalizability of the findings to other regions or seasons, emphasizing the need for additional studies. The complexity of natural extracts poses a challenge in understanding the role of individual compounds/fractions in their anti-inflammatory activity, which could limit the ability to draw definitive conclusions. While some studies identified correlations between certain compounds and various outcomes, the precise molecular mechanisms and specific pathways underlying the observed effects are not fully understood. The absence of clinical trials highlights a gap in translating these findings to real-world applications and emphasizes the necessity for future clinical investigations.

## 7. Conclusions

In conclusion, this comprehensive literature review explored the anti-inflammatory properties of brown algae as well as the multifaceted therapeutic potential of brown algae. In particular, key compounds such as fucoidans and polyphenols have been thoroughly looked at. These compounds demonstrate notable anti-inflammatory effects and these qualities can be harnessed for the development of dermatological formulations. Advanced extraction techniques and innovative delivery systems seem to hold potential in optimizing the bioavailability and efficacy of these compounds for future dermatological applications. While in vivo trials show promising outcomes, further studies and robust clinical trials are essential for assessing safety and efficacy for future practical applications. In order to fully extract the therapeutic potential of brown algae and enable their use in dermatological formulations, future research should concentrate on identifying the precise mechanisms of action, isolating bioactive components, refining extraction methods and carrying out clinical trials. This review marks a significant step toward the development of all-natural skincare treatments, highlighting the need for continued research to fully realize the potential of brown algae in treating inflammatory skin conditions.

## Figures and Tables

**Table 1:** List of brown algae species investigated.

Brown Algae Species Investigated	Chemical Compounds Investigated
*Fucus vesiculosus*	Fucoidan
*Sargassum wightii*	Sulphated polysaccharides
*Saccharina japonica*	Fucoidan
*Fucus distichus*	Fucoidan
*Fucus serratus*	Fucoidan
*Ascophyllum nodosum*	Fucoidan
*Dictyota menstrualis*	Heterofucan
*Saccorhiza polyschides*	Not specified
*Sargassum siliquosum*	Fucoidan
*Carpomitra costata*	Not specified
*Sargassum vulgare*	Phlorotannins
*Ecklonia maxima*	Fucoidan
*Sargassum ilicifolium*	Not specified
*Polycladia crinita*	Polysaccharides, lipids, terpenoids, fucosterol, phlorotannins
*Cystoseira crinita*	Fucoidan
*Cystoseira compressa*	Fucoidan
*Cystoseira sedoides*	Fucoidan
*Halopteris scoparia* (Linnaeus) Sauvageau	Not specified
*Sargassum swartzii*	Not specified
*Cystoseira indica*	Not specified
*Codium flabellatum*	Not specified
*Cystoseira sedoides* (Fucales)	Fucoidan
*Cladostephus spongeosis*	Phlorotannins
*Padina pavonica*	Phlorotannins
*Padina boergesenii*	Chlorophylls, carotenoids, fucoxanthin, phycoerythrin, phycocyanin, lycopene, amino acids, polyphenols, proteins
*Halopteris scoparia*	Not specified

**Table 2:** Study year, algae species and compounds, study design, anti-inflammatory assays and outcome measures of the included studies.

Title	Year	Algae Species and Compounds	Study Design	Anti-Inflammatory Assays	Outcome Measures
In Vitro Anti-Inflammatory Activities of Fucoidans from Five Species of Brown Seaweeds	2022	Algae species: *Saccharina japonica*, *Fucus vesiculosus*, *Fucus distichus*, *Fucus serratus*, and *Ascophyllum nodosum*. Compounds: Fucoidan	In vitro	1. 1,1-diphenyl-2-picrylhydrazyl (DPPH) radical scavenging assay	This study measures the anti-inflammatory outcomes of the fucoidans through: 1. Their ability to scavenge free radicals 2. Inhibit protein denaturation.
Evaluation of anti-nociceptive and anti-inflammatory activities of a heterofucan from *Dictyota menstrualis*	2013	Algae species: *Dictyota menstrualis* Compounds: Heterofucan	In vivo	1. Assessment of pro-inflammatory cytokines, including interleukin-1 beta (IL-1β), interleukin-6 (IL-6), and tumor necrosis factor alpha (TNF-α)	This study measured the impact of *Dictyota menstrualis* on: 1. Leukocyte migration into the peritoneal cavity after chemical stimulation 2. Its effect on the expression of pro-inflammatory cytokines
Saccorhiza polyschides—A Source of Natural Active Ingredients for Greener Skincare Formulations	2022	Algae species: *Saccorhiza polyschides* Compounds: No specific compound	In vitro	1. Enzyme-linked immunosorbent assay (ELISA) to determine the levels of pro-inflammatory cytokines (TNF-α and IL-6) and anti-inflammatory cytokine (IL-10) in RAW 264.7 cells 2. 1,1-Diphenyl-2-picrylhydrazyl (DPPH) radical scavenging assay	1. Measurement of pro-inflammatory and anti-inflammatory cytokines 2. Inhibition of nitric oxide (NO) production 3. Cell viability and cytotoxicity assessment 4. Reactive oxygen species (ROS) production
Fucoidan from seaweed *Fucus vesiculosus* inhibits 2,4-dinitrochlorobenzene-induced atopic dermatitis	2019	Algae species: *Fucus vesiculosus* Compounds: Fucoidan	In vitro	1. Ear Swelling and Erythema Score 2. Histology (hematoxylin and eosin staining) 3. Immunofluorescence staining for scavenger receptor class A (SR-A) and CD4+ T cells in ear tissues 4. Enzyme-linked immunosorbent assay (ELISA) to measure serum levels of immunoglobulin E (IgE) and interleukin-4 (IL-4) 5. Real-time PCR for quantification of mRNA levels of SR-A, pro-inflammatory cytokines (TNF-α, IL-12), anti-inflammatory cytokines (IL-4, IL-10, TGF-β), and GAPDH (housekeeping gene) 6. Spleen Index calculation of spleen weight relative to body weight 7. Cell proliferation assay to evaluate splenocyte proliferation using the Cell Counting Kit-8 (CCK-8) 8. Flow cytometry: analysis of CD4+ T cell subsets, including Th1, Th2, Th17, and regulatory T cells (Treg).	Primary outcome measures included the assessment of: 1. Ear swelling 2. Skin lesions 3. Inflammatory cell infiltration Secondary outcomes included the measurement of: 1. Serum levels of IgE and IL-4 2. Histological analysis of ear tissues 3. Evaluation of spleen index 4. Splenocyte proliferation 5. Characterization of CD4+ T cell subsets
Effect of molecular mass and sulphate content of fucoidan from *Sargassum siliquosum* on antioxidant, anti-lipogenesis, and anti-inflammatory activity	2021	Algae species: *Sargassum siliquosum* Compounds: Fucoidan	In vitro	1. Assessment of the proinflammatory cytokine TNF-α production in RAW264.7 cells	The primary outcome measures include the antioxidant capacity, anti-lipogenesis activity, and anti-inflammatory activity, which were evaluated based on: 1. Scavenging effect on DPPH radicals (antioxidant) 2. Inhibition of lipid synthesis in HepG2 cells (anti-lipogenesis), 3. Reduction of TNF-α production in RAW264.7 cells (anti-inflammatory). The effects were compared among different fucoidan fractions with varying molecular masses and sulphate contents.
Unravelling the Dermatological Potential of the Brown Seaweed *Carpomitra costata*	2021	Algae species: *Carpomitra costata* Compounds: No specific compound	In vitro	1. 1,1-Diphenyl-2-picrylhydrazyl (DPPH) radical scavenging assay 2. Assessment of anti-inflammatory cytokines (TNF-α, IL-6, and IL-10) after mouse macrophage cells (RAW 264.7) exposure to lipopolysaccharide (LPS) as an inflammation mediator	1. Production of nitric oxide (NO) in mouse macrophage cells 2. The response to lipopolysaccharide (LPS) exposure 3. The levels of inflammatory and anti-inflammatory cytokines (TNF-α, IL-6, and IL-10) The results are expressed as a percentage of the control or in terms of inhibitory effects.
Phlorotannins of the Brown Algae *Sargassum vulgare* from the Mediterranean Sea Coast	2022	Algae species: *Sargassum vulgare* Compounds: Phlorotannins	In vitro	1. Inhibition of albumin denaturation	1. Inhibition of albumin denaturation serves as the outcome measure for the anti-inflammatory activity, and the effectiveness of different fractions 2. Assessing the ability of the samples to scavenge free radicals and prevent oxidation 3. Characterization of phlorotannins within the ethyl acetate fraction, identifying various compounds and derivatives based on LC-ESI-MS/MS analysis
Anti-Fine Dust Effect of Fucoidan Extracted from *Ecklonia maxima* Laves in Macrophages via Inhibiting Inflammatory Signalling Pathways	2022	Algae species: *Ecklonia maxima* Compounds: Fucoidan	In vitro	1. MTT test for cell viability assessment of particulate matter stimulated RAW 264.7 macrophages 2. Griess assay for the determination of NO production in particulate matter stimulated cells 3. ELISA assay for measuring PGE2 production in particulate matter stimulated RAW 264.7 macrophages 4. Competitive enzyme immunoassay kits for quantifying TNF-α, IL-6, and IL-1β concentrations in the supernatant 5. Western blot analysis for evaluating protein expression levels of iNOS, COX-2, NF-κB, and MAPK in particulate matter stimulated RAW 264.7 cells 6. RT-qPCR for assessing pro-inflammatory cytokine expression levels (iNOS, COX2, IL-1β, IL-6, TNF-α, TLR2, and TLR4) in *E. maxima* fucoidan-treated RAW 264.7 cells.	The inhibitory effects of E. maxima fucoidan on the: 1. Production of NO 2. Production of PGE2 3. Production of pro-inflammatory cytokines The impact of *E. maxima* fucoidan on the expression of iNOS, COX-2, NF-κB, and MAPK proteins.
Antinociceptive and anti-inflammatory effect of sulphated polysaccharide fractions from *Sargassum wightii* and *Halophila ovalis* in male Wistar rats	2016	Algae species: *Sargassum wightii* Compounds: sulphated polysaccharides	In vivo	1. Carrageenan-induced rat paw oedema 2. Peritonitis model in rats 3. Freund’s adjuvant-induced arthritis	1. Reduction in paw volume variation over time 2. Decrease in total leukocyte count in peritoneal fluid 3. Percentage of inhibition of arthritis development
Evaluation of marine brown algae *Sargassum ilicifolium* extract for analgesic and anti-inflammatory activity	2013	Algae species: *Sargassum ilicifolium* Compounds: No specific compound	In vivo	1. Carrageenan-induced rat paw oedema	1. Reduction in paw swelling over time, expressed as a percentage of inhibition compared to control
New investigation of anti-inflammatory activity of Polycladia crinita and biosynthesized selenium nanoparticles: isolation and characterization	2023	Algae species: *Polycladia crinita* Compounds: polysaccharides, lipids, terpenoids, fucosterol, and phlorotannins	In vivo	1. Carrageenan-induced rat paw oedema	1. Measurement of paw oedema weight changes 2. Tissue levels of malondialdehyde (MDA) and prostaglandin E2 (PGE2) 3. Histopathological examination of paw tissues 4. Immunohistochemical staining for COX-2 and IL-1β expression in paw tissues
Physico-chemical characterization and pharmacological evaluation of sulphated polysaccharides from three species of Mediterranean brown algae of the genus *Cystoseira*	2015	Algae species: *Cystoseira crinita*, *Cystoseira compressa*, and *Cystoseira sedoides* Compounds: Fucoidan	In vivo	1. Carrageenan-induced rat paw oedema 2. 1,1-Diphenyl-2-picrylhydrazyl (DPPH) radical scavenging assay	1. The reduction in carrageenan-induced paw oedema over time 2. EC50 value, representing the effective concentration at which the antioxidant activity was 50%
Marine endophytic fungi associated with *Halopteris scoparia* (Linnaeus) Sauvageau as producers of bioactive secondary metabolites with potential dermocosmetic application	2021	Algae species: *Halopteris scoparia* (Linnaeus) Sauvageau Compounds: No specific compound	In vitro	1. MTT assay was used to measure the amount of nitric oxide produced by RAW 264.7 cells incubated in various concentrations of algae extract 2. 1,1-Diphenyl-2-picrylhydrazyl (DPPH) radical scavenging assay	1. Measuring the production of nitric oxide (NO) in RAW 264.7 cells in response to LPS exposure and evaluate for their ability to suppress NO production
Phycochemical analyses and pharmacological activities of seven macroalgae of Arabian Sea (Northern coast line)	2021	Algae species: *Sargassum wightii*, *Sargassum swartzii*, *Cystoseira indica*, and *Codium flabellatum* (*other non brown algae species were also investigated) Compounds: No specific compounds	In vivo	1. Carrageenan-induced rat paw oedema	1. Measurement of paw size at specific time points (1st, 2nd, 3rd, 4th, and 5th hours) after the induction of inflammation using the carrageenan suspension
Phytochemical Analysis and Evaluation of the Antioxidant, Anti-Inflammatory, and Antinociceptive Potential of Phlorotannin-Rich Fractions from Three Mediterranean Brown Seaweeds	2018	Algae species: *Cystoseira sedoides* (Fucales), *Cladostephus spongeosis* (Sphacelariales), and *Padina pavonica* (Dictoyales) Compounds: Phlorotannins	In vivo	1. Xylene-induced ear oedema in mice 2. Carrageenan-induced paw oedema in rats	1. Inhibition percentage of ear oedema in the xylene-induced ear oedema model 2. The percentage of inhibition of paw oedema in the carrageenan-induced paw oedema model
Comprehensive Phytochemical Analysis and Bioactivity Evaluation of *Padina boergesenii*: Unveiling Its Prospects as a Promising Cosmetic Component	2023	Algae species: *Padina boergesenii* Compounds: Chlorophylls, carotenoids, fucoxanthin, phycoerythrin, phycocyanin, lycopene, amino acids, polyphenols, and proteins	In vitro	1. No specific anti-inflammatory assay was used (*the study identified compounds within algae with known anti-inflammatory effects)	1. Identification of bioactive compounds with potential cosmetic benefits
Primary structural features, physicochemical and biological properties of two water-soluble polysaccharides extracted from the brown Tunisian seaweed *Halopteris scoparia*	2023	Algae species: *Halopteris scoparia* Compounds: Alginates and fucoidans	In vitro	1. Denaturation of bovine serum albumin (BSA) induced by heat	1. The inhibition of albumin denaturation was calculated

## Data Availability

The data supporting the findings of this study were obtained from articles available on PubMed. Specific details and datasets analysed during the study can be accessed through the respective articles cited in the references. No new data were created in this study. Data availability is subject to the privacy and ethical policies of the original publications.
